# Suppressive role of E3 ubiquitin ligase FBW7 in type I diabetes in non-obese diabetic mice through mediation of ubiquitination of EZH2

**DOI:** 10.1038/s41420-021-00605-x

**Published:** 2021-11-20

**Authors:** Yingxue Guo, Junfeng Li, Shuang Fan, Qibo Hu

**Affiliations:** 1 Department of Clinical Laboratory, The Second Hospital of Jilin University, Changchun, 130041 P. R. China; 2 Department of Gastroenterology, The Second Hospital of Jilin University, Changchun, 130041 P. R. China; 3 Department of Pediatrics, The Second Hospital of Jilin University, Changchun, 130041 P. R. China

**Keywords:** Biochemistry, Diseases

## Abstract

The current study tried to uncover the molecular mechanism of E3 ubiquitin ligase F-box and WD repeat domain-containing 7 (FBW7) in a heritable autoimmune disease, type I diabetes (T1D). After streptozotocin-induced T1D model establishment in non-obese diabetic (NOD) mouse, the protein expression of FBW7, enhancer of zeste homolog 2 (EZH2), and Zinc finger and BTB domain containing 16 (ZBTB16) was quantified. Next, splenocytes and pancreatic beta cells were isolated to measure the production of pro-inflammatory cytokines in splenocytes, as well as islet beta-cell apoptosis. Additionally, the stability of EZH2 induced by FBW7 was analyzed by cycloheximide chase assay. The binding affinity of FBW7 and EZH2 and the consequence of ubiquitination were monitored by co-immunoprecipitation assay. Last, a chromatin immunoprecipitation assay was employed to analyze the accumulation of EZH2 and H3K27me3 at the ZBTB16 promoter region. Our study demonstrated downregulated FBW7 and ZBTB16 and upregulated EZH2 in diabetic NOD mice. Overexpression of FBW7 in the NOD mice inhibited pro-inflammatory cytokine release in the splenocytes and the apoptosis of islets beta cells. FBW7 destabilized EZH2 and accelerated ubiquitin-dependent degradation. EZH2 and H3K27me3 downregulated the ZBTB16 expression by accumulating in the ZBTB16 promoter and methylation. FBW7 upregulates the expression of ZBTB16 by targeting histone methyltransferase EZH2 thus reducing the occurrence of T1D.

## Introduction

Type I diabetes (T1D) is a heritable autoimmune disease [1]. Caused by an autoimmune response against pancreatic beta cells, T1D results in beta cell destruction and may accompany another autoimmune disease [2]. T1D is generally considered a juvenile-onset disease but it can occur at any age of life. Patients have to administrate insulin for survival causing a tremendous burden to individuals and society psychologically and economically [3]. T1D is a chronic autoimmune disease, which seriously impairs the pancreatic beta cells [4]. Healthcare providers are struggling for economic and standardized therapies.

F-box and WD repeat domain-containing 7 (FBW7, also known as FBXW7), an E3 ubiquitin ligase, is found to be a pancreatic beta-cell apoptosis inhibitor in vivo [5]. Of note, a recent study has suggested that FBW7 prevents insulin resistance in the type 2 diabetes (T2D) mouse models [6]. Despite that previous genome-wide association studies (GWAS) showing FBW7 contributed to the risk for T2D [7], evidence for the association of FBW7 and T1D is limited. These results highlight the implication of FBW7 in diabetes and encourage us to investigate the functional relevance of FBW7 in T1D.

As a potential substrate of FBW7, enhancer of zeste homolog 2 (EZH2) transfers a methyl group from *S*-adenosyl-l-methionine to the lysine 27 on histone H3 [8]. Methylation by EZH2 assists the heterochromatin formation and suppresses the respective gene [9]. Interestingly, prior evidence has suggested that EZH2 is able to modulate pancreatic beta-cell proliferation and regeneration in diabetes mellitus [10]. A recent report has documented that EZH2 can be suppressed by FBW7 in pancreatic cancer cells, thus restraining tumor cell migration and invasion [11]. Taken together, we reasonably propose EZH2 interacts with FBW7 and pancreatic cells in diabetes, which would fill the gap of FBW7 in T1D.

Zinc finger and BTB domain containing 16 (ZBTB16, also known as PLZF), initially discovered as a cause of human retinoic acid-resistant acute promyelocytic leukemia, is a DNA sequence-specific transcriptional repressor [12]. Increasing studies have highlighted that ZBTB16 expression correlates with diabetes through the adaptive thermogenesis response, mitochondrial respiration promotion [13], and regulation of the insulin signaling pathway [14]. Importantly, ZBTB16 is found to be a possible candidate therapeutic target for T1D [15].

Here, we report that a regulatory axis of the three factors, FBW7, EZH2, and ZBTB16 contributes to the reduction of T1D occurrence. In this pathway, FBW7 degrades EZH2 and upregulates ZBTB16. We show that FBW7 is an emergent new therapeutic target for T1D treatment.

## Results

### FBW7 is under-expressed in a mouse model with T1D

**Fig. 1:**
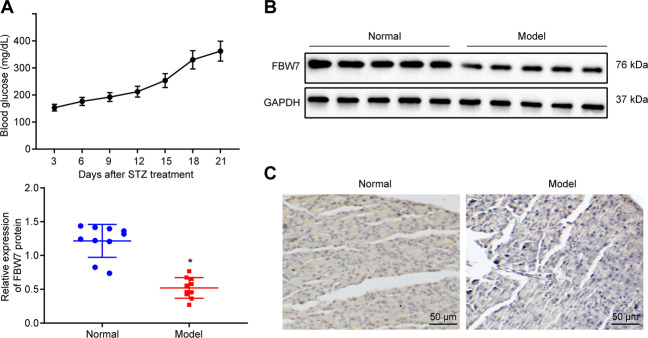
FBW7 is under-expressed in the T1D mouse model. **A** The blood glucose level in the mouse model. **B** Western blot results of FBW7 expression in islets from T1D mice (*n* = 10) and control mice (*n* = 10), *p < 0.05, vs. the normal group. **C** Representative images of IHC for FBW7 expression in islets from T1D mice and control mice with arrows pointing at cells positive for FBW7 (bar = 25 μm). Measurement data are presented. Data comparisons between two groups were analyzed by unpaired *t*-test. Sta*t*istical analysis in relation to time-based measurements within each group was realized using repeated-measures ANOVA.

The correlation between FBW7 and diabetic nephropathy has been previously established [16]. The T1D mouse model was established using streptozotocin (STZ). After 15 days of STZ administration, averaged blood glucose levels of the modeled mice exceeded 250 mg/dL, which confirmed the successful establishment of T1D mouse models (Fig. 1A). FBW7 expression levels in the pancreatic islets of mice were examined by Western blot assay and immunohistochemistry (IHC). A significant decrease of the FBW7 expression level was observed in the T1D model group (Fig. 1B and C). The above results concluded that FBW7 was downregulated in the T1D mouse model.

### FBW7 suppresses the occurrence of T1D in vivo and in vitro

**Fig. 2:**
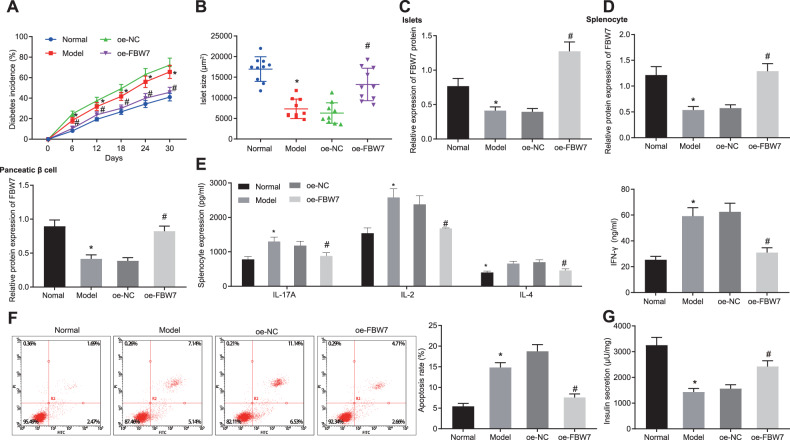
FBW7 decreases the occurrence of T1D in vivo and in vitro. **A** The diabetes incidence of each group. **B** The islet size of each group. **C** FBW7 expression in mice islet of each group determined by Western blot analysis. **D** FBW7 expression in induced splenocytes and pancreatic beta cells of each group was determined by Western blot analysis. **E** The levels of IL-17A, IL-2, IFN-γ, and IL-4 in cell supernatant of each group were determined by ELISA. **F** The pancreatic beta-cell apoptosis of each group was determined by flow cytometry. **G** The insulin secretion of each group. *p < 0.05, vs. the normal group, ^#^*p* < 0.05, vs. the OE-NC group. All experiments were conducted in triplicate. Data comparisons between two groups were analyzed by unpaired *t*-test. Comparisons among multiple groups were performed by one-way ANOVA. Statistical analysis in relation to time-based measurements within each group was realized using repeated-measures ANOVA.

To further illustrate the impact of FBW7 on T1D, we overexpressed FBW7 in the T1D mouse model and analyzed the incidence of T1D in both groups. Increased T1D incidence was observed in modeled mice. Besides, T1D incidence declined noticeably in the presence of overexpression FBW7 (OE-FBW7) (Fig. 2A). The size of the islet was measured after isolation. Results showed that mice islets were remarkably shrunken in the model group in comparison with the normal group while islets were expanded in the OE-FBW7 group as to the overexpression negative control (OE-NC) group (Fig. 2B). FBW7 expression determination using Western blot analysis revealed decreased FBW7 expression in the islet from modeled mice. The expression level of FBW7 in islets was elevated in the presence of OE-FBW7 (Fig. 2C). Therefore, we showed FBW7 decreased the T1D incidence in vivo.

Autoreactive T cells are considered as the major factor in beta cell destruction [4]. Therefore, we simulated the T1D-induced islets by mixing splenocytes and T1D-specific T cells and induced Min6 cell apoptosis in beta cells using inflammatory factor tumor necrosis factor-α (TNF-α). FBW7 was overexpressed in a part of cells from modeled mice. FBW7 expression in the splenocytes and pancreatic beta cells was checked by Western blot analysis. We observed that FBW7 in both cell types from modeled mice was downregulated. Besides, OE-FBW7 treatment upregulated FBW7 expression in both cell types (Fig. 2D). Enzyme-linked immunosorbent assay (ELISA) was applied to measure the interleukin-17 (IL-17), IL-2, interferon-γ (IFN-γ), and IL-4 levels in the cell supernatant (Fig. 2E) with the findings revealed that levels of IL-17, IL-2, IFN-γ, and IL-4 elevated in modeled mice while those were reduced in the presence of OE-FBW7. These results demonstrated FBW7 could eliminate the activation of autoreactive T cells thus preventing T1D.

Additionally, pancreatic beta-cell apoptosis verified by flow cytometry showed accelerated apoptosis in modeled mice, while apoptosis decreased after OE-FBW7 treatment (Fig. 2F). Further, we found lessened insulin secretion in modeled mice which was enhanced in the presence of OE-FBW7 (Fig. 2G). The aforementioned results demonstrated that FBW7 decreased the T1D incidence both in vitro and in vivo.

### FBW7 inhibits TNF-α-induced pancreatic beta-cell apoptosis and dysfunction through ubiquitin degradation of EZH2

T1D downstream pathway affected by FBW7 was further investigated. Researchers reported that EZH2, highly expressed in T1D rats [17], was a substrate of FBW7 and negatively regulated [11].

**Fig. 3:**
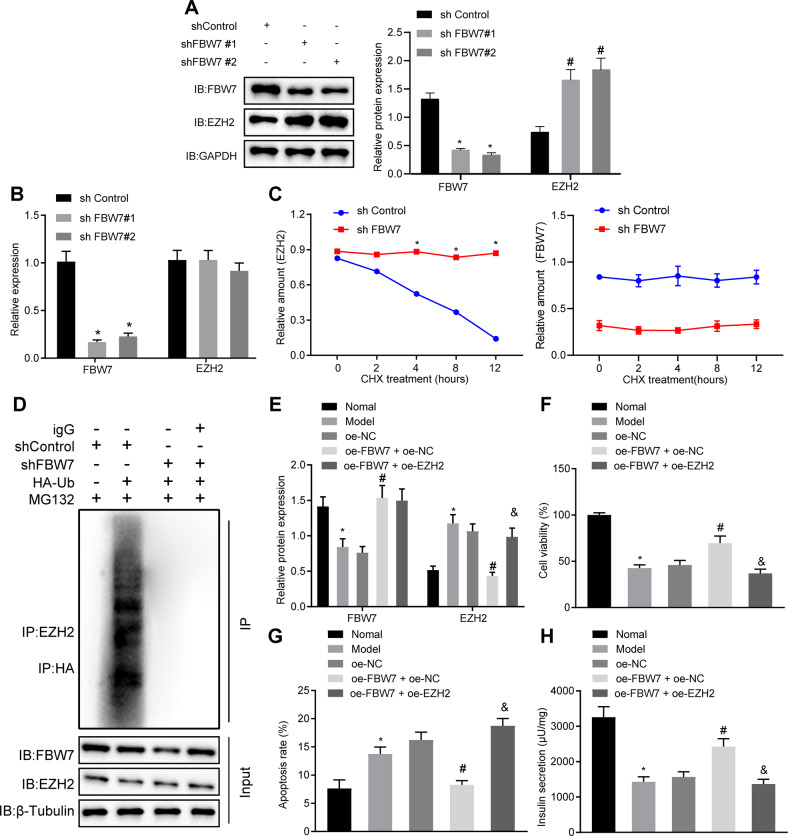
FBW7 inhibits TNF-α-induced pancreatic beta-cell apoptosis and dysfunction through ubiquitin degradation of EZH2. **A** Western blot assay of Min6 cells treated with MG132, **B** Western blot assay of Min6 cells transfected with shFBW7, *p < 0.05 vs. the shControl group in FBW7 blotting, ^#^*p* < 0.05 vs. the shControl group in EZH2 blotting. **C** RT-qPCR quantification of FBW7 and EZH2 mRNA in Min6 cells transfected with shFBW7, *p < 0.05 vs. the shControl group. **D** Western blot analysis of EZH2 after CHX treatment at different time points, EZH2 was first normalized to GAPDH then normalized to time 0 at each time point, *p < 0.05 vs. the shControl grou*p*. **E** After 48 h of transfection of designed plasmid, the Min6 cells were incubated with the MG132 (20 μM) for 8 h, Western blot analysis of the EZH2 ubiquitination following with IP assay. **F** Western blot assay of the FBW7 and EZH2 proteins in the FBW7-, or with EZH2-, overexpressed Min6 cells, *p < 0.05 vs. the normal group, ^#^*p* < 0.05 vs. the OE-EZH2 + OE-NC group, ^&^*p* < 0.05 vs. the OE-EZH2 + OE-FBW7 group. **G** CCK-8 assay for cell viability in each group, *p < 0.05 vs. the normal group, ^#^*p* < 0.05 vs. the OE-EZH2 + OE-NC group, ^&^*p* < 0.05 vs. the OE-EZH2 + OE-FBW7 group. **H** flow cytometry determination for apoptosis in each group, *p < 0.05 vs. the normal group, ^#^*p* < 0.05 vs. the OE- EZH2 + OE-NC group, ^&^*p* < 0.05 vs. the OE-EZH2 + OE-FBW7 group. **I** Determination of insulin secretion, *p < 0.05 vs. the normal group, ^#^*p* < 0.05 vs. the OE-EZH2 + OE-NC group, ^&^*p* < 0.05 vs. the OE-EZH2 + OE-FBW7 group. All experiments were conducted in triplicate. Data comparisons between two groups were analyzed by unpaired *t*-test. Comparisons among multiple groups were performed by one-way ANOVA. Statistical analysis in relation to time-based measurements within each group was realized using repeated-measures ANOVA.

To clarify whether FBW7 acted as E3 ubiquitin ligase of EZH2, we transfected the Myc-tagged FBW7 vector in gradually increased concentration or empty vector, and Flag-tagged EZH2 vector at a constant concentration into Min6 cells. After 24 h, the cells were incubated with MG132, a proteasome inhibitor, or dimethyl sulfoxide for 16 h. Western blot analysis revealed that ectopically expressed EZH2 was downregulated by FBW7 while under-expression of EZH2 was blocked by MG132 (Fig. 3A). Additionally, knocking down the endogenous FBW7 by two independent *Fbw7*-specific short hairpin RNAs (shRNAs) enhanced the EZH2 protein expression level (Fig. 3B), leaving EZH2 mRNA expression unchanged (Fig. 3C). Cycloheximide (CHX), an inhibitor of eukaryotic translation, was added to observe the effect of FBW7 on EZH2 expression after CHX addition. According to Western blot analysis, EZH2 translation was significantly inhibited over the prolong of CHX treatment and endogenous EZH2 protein level was significantly reduced. Moreover, knocking down *Fbw7* increased the stability of endogenous EZH2 (Fig. 3D). Results obtained from co-IP demonstrated EZH2 polyubiquitination was diminished as *Fbw7* was knocked down (Fig. 3E). The results above indicated that FBW7 boosted EZH2 ubiquitination and proteasome degradation.

Next, we explained the effect of FBW7 and EZH2 on the functions of TNF-α-induced pancreatic beta cells. Min6 cells were treated with OE-FBW7 or OE-EZH2. As shown in Fig. 3F, FBW7 was upregulated while EZH2 was downregulated in the presence of OE-FBW7, which was then remedied by EZH2 overexpression. In addition, higher cell viability and less apoptosis were observed in the presence of OE-FBW7 while overexpression EZH2 simultaneously brought about more cytotoxicity and accelerated apoptosis (Fig. 3G, H). Assessment of glucose-induced insulin secretion proved that more insulin was secreted in the presence of OE-FBW7 while decreased by additional EZH2 overexpression (Fig. 3I). Thus, TNF-α-induced beta cells apoptosis and dysfunction were inhibited by FBW7 through ubiquitin degradation of EZH2.

### EZH2 enhances TNF-α-induced pancreatic beta cells apoptosis and dysfunction through ZBTB16 methylation

**Fig. 4:**
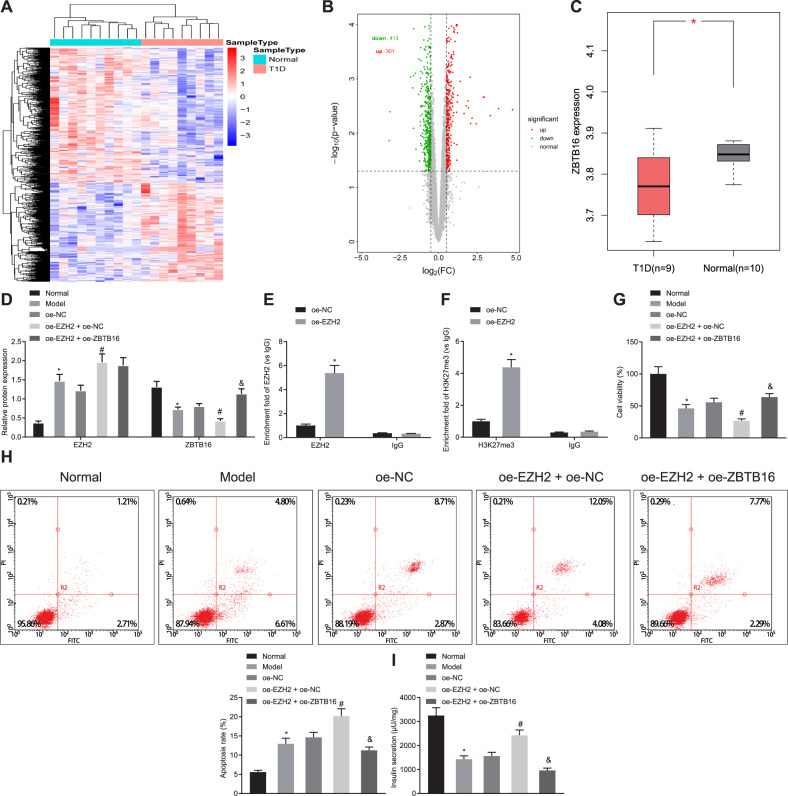
EZH2 enhances TNF-α-induced pancreatic beta-cell apoptosis and dysfunction through ZBTB16 methylation. **A** Clustering heat map of differentially expressed genes in a T1D-related dataset, each row represents a gene, and each column represents a sample. **B** Volcano map of differentially expressed genes in T1D-related dataset, each dot represents a gene, where red dots indicate up-regulation and green dots indicate down-regulation. **C** The expression of ZBTB16 in the dataset, red indicates T1D samples, gray indicates normal tissue samples. **D** Western blot assay of the EZH2 and ZBTB16 proteins in the EZH2-, or with ZBTB16-, overexpressed Min6 cells, *p < 0.05 vs. the normal group, ^#^*p* < 0.05 vs. the OE-EZH2 + OE-NC group, ^&^*p* < 0.05 vs. the OE-EZH2 + OE-ZBTB16 group. E ChIP assay for detecting EZH2 accumulation in ZBTB16 promoter, *p < 0.05 vs. the OE-NC group. **F** ChIP assay for detecting H3K27me3 accumulation in ZBTB16 promoter, *p < 0.05 vs. the OE-NC group. **G** CCK-8 assay for cell variability, *p < 0.05 vs. the normal group, ^#^*p* < 0.05 vs. the OE-EZH2 + OE-NC group, ^&^*p* < 0.05 vs. the OE-EZH2 + OE-ZBTB16 group. **H** Flow cytometry assay for apo*p*tosis, *p < 0.05 vs. the normal group, ^#^*p* < 0.05 vs. the OE-EZH2 + OE-NC group, ^&^*p* < 0.05 vs. the OE-EZH2 + OE-ZBTB16 group. **I** Determination of insulin secretion, *p < 0.05 vs. the normal group, ^#^*p* < 0.05 vs. the OE-EZH2 + OE-NC group, ^&^*p* < 0.05 vs. the OE-EZH2 + OE-ZBTB16 grou*p*. All experiments were conducted in triplicate. Data comparisons between two groups were analyzed by unpaired *t*-test. Comparisons among multiple groups were performed by one-way ANOVA.

The R language limma software package was used for differential analysis, and the clustering heat map of differentially expressed genes was drawn (Fig. 4A). A total of 714 significantly differentially expressed genes were screened, of which 301 were up-regulated and 413 were down-regulated (Fig. 4B). Among them, ZBTB16 expression was down-regulated in T1D samples (Fig. 4C). It has been reported that EZH2 suppresses ZBTB16 expression [18]. Min6 cell model was established by EZH2 overexpression, or with ZBTB16 overexpression simultaneously, and TNF-α induction. Western blot analysis showed that OE-EZH2 led to increased EZH2 but reduced ZBTB16, while overexpression of ZBTB16 reversed these changes (Fig. 4D). Considering the possibility of *Zbtb16* methylation, chromatin immunoprecipitation (ChIP) assay demonstrated that EZH2 and EZH2-mediated trimethylation of lysine 27 on histone 3 (H3K27me3) were accumulated in the *Zbtb16* promoter region (Fig. 4E, F).

Furthermore, analysis of cell viability and apoptosis indicated that OE-EZH2 inhibited cell viability but accelerated cell apoptosis, while further addition of OE-ZBTB16 reversed the above changes (Fig. 4G, H). Glucose-induced insulin secretion assay proved that insulin secreted more in the presence of OE-EZH2 while insulin secretion was eliminated by further addition of OE-ZBTB16 (Fig. 4I). Therefore, we were confident to conclude that EZH2 mediated *Zbtb16* methylation thus enhancing TNF-α-induced pancreatic beta cells apoptosis and dysfunction.

### FBW7 inhibits T1D occurrence via the EZH2/ZBTB16 axis in vivo and in vitro

**Fig. 5:**
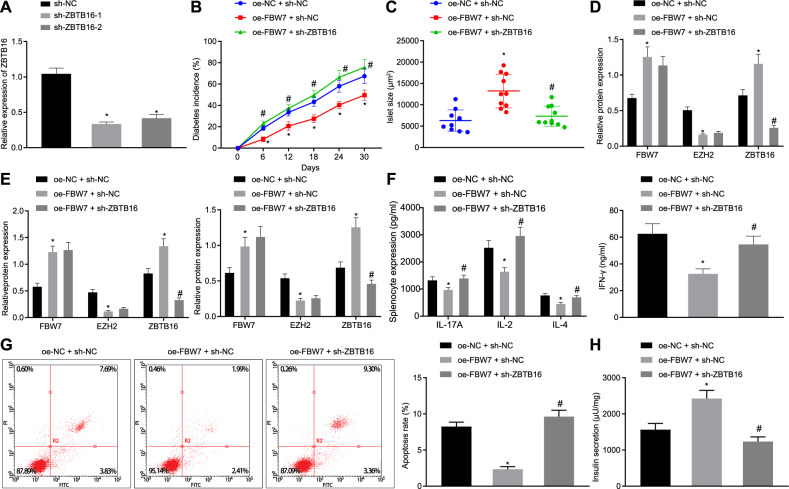
FBW7 inhibits T1D occurrence via EZH2/ZBTB16 in vivo and in vitro. **A** RT-qPCR detection of ZBTB16 mRNA level in each group, *p < 0.05 vs. the OE-NC group. **B** The diabetes incurrence in each mouse model group, *p < 0.05 vs. the OE-FBW7 + sh-NC group. **C** The islet size in each mice model group, *p < 0.05 vs. the OE-FBW7 + sh-NC group. **D** Western blot detection of FBW7, EZH2, and ZBTB16 expression in islet, *p < 0.05 vs. the OE-FBW7 + sh-NC group, ^#^*p* < 0.05 vs. the OE-FBW7 + sh-NC group. **E** Western blot detection of FBW7, EZH2, and ZBTB16 expression in splenocytes and pancreatic beta cells, *p < 0.05 vs. the OE-FBW7 + sh-NC group, ^#^*p* < 0.05 vs. the OE-FBW7 + sh-NC group. **F** ELISA detection of IL-17A, IL-2, IFN-γ, and IL-4 in cell supernatant, *p < 0.05 vs. the OE-FBW7 + sh-NC group. **G** Flow cytometry detection of pancreatic beta-cell apoptosis in each group, *p < 0.05 vs. the OE-FBW7 + sh-NC group; H) Determination of insulin secretion, *p < 0.05, vs. the OE-FBW7 + sh-NC group. All experiments were conducted in triplicate. Comparisons among multiple groups were performed by one-way ANOVA. Statistical analysis in relation to time-based measurements within each group was realized using repeated-measures ANOVA.

To verify the role of FBW7/EZH2/ZBTB16 axis in T1D, we transfected the sh-ZBTB16 vector to the Min6 cells, and the silencing efficiency was confirmed by reverse transcription-quantitative polymerase chain reaction (RT-qPCR) with sh-ZBTB16-1 showed the best efficiency selected in the following experiments (Fig. 5A). T1D mouse models with *Fbw7* overexpression, or simultaneously with *Zbtb16* suppression using shRNA were established and checked for diabetes incidence (Fig. 5B). Compared to the OE-FBW7 + sh-NC group, the OE-FBW7 + sh-ZBTB16 group showed a significantly higher occurrence of T1D. Then, measurement of islets showed that islets from the OE-FBW7 + sh-ZBTB16 group shrank considerably referred to as the OE-FBW7 + sh-NC group (Fig. 5C). Western bolt analysis was done to determine the expression of FBW7, EZH2, and ZBTB16 in the islet (Fig. 5D). Expression levels of FBW7 and ZBTB16 were escalated while EZH2 declined in the OE-FBW7 + sh-NC group as to the OE-NC group. In comparison to the OE-FBW7 + sh-NC group, no significant change in FBW7 and EZH2 expression but a reduction in ZBTB16 expression was observed.

Meanwhile, expression of FBW7, EZH2, and ZBTB16 in the splenocytes and pancreatic beta cells were revealed by Western blot analysis (Fig. 5E). Compared to the OE-NC + sh-NC group, FBW7 and ZBTB16 were elevated while EZH2 was reduced in the OE-FBW7 + sh-NC group. Referring to the OE-FBW7 + sh-NC group, expression of FBW7 and EZH2 stayed consistent while ZBTB16 decreased in the OE-FBW7 + sh-ZBTB16 group. Quantification of IL-17, IL-2, IFN-γ and IL-4 in the cell supernatant by ELISA showed that all four cytokines were boosted in the OE-FBW7 + sh-ZBTB16 group compared to the OE-FBW7 + sh-NC group (Fig. 5F). Determination of pancreatic beta cell apoptosis by flow cytometry indicated expedited apoptosis occurred in the OE-FBW7 + sh-ZBTB16 group referring to the OE-FBW7 + sh-NC group (Fig. 5G). Assessment of glucose-induced insulin secretion confirmed that less insulin was secreted in the OE-FBW7 + sh-ZBTB16 group as to the OE-FBW7 + sh-NC group (Fig. 5H). In conclusion, FBW7 inhibited T1D occurrence via the EZH2/ZBTB16 pathway both in vivo and in vitro.

## Discussion

In the past decades, our knowledge on T1D increased rapidly, but insufficient to satisfy the demands on standardized clinical treatment and contracting disease-associated burdens. Researches on T1D were concentrated on the mechanism of autoimmunity [19] and genetically relevant loci in human leukocyte antigen (HLA) [20, 21], which might benefit T1D prediction and prevention [22]. Here in the present study, the mechanism of EZH2/ZBTB16 in T1D inhibition by FBW7 is clarified, suggesting that FBW7 is a promising target for T1D therapy.

Previous GWAS showed that more than 40 loci link to the risk of T1D [23], a few of which are non-HLA loci, e.g. INS, CTLA4, and PTPN22. We are interested in these non-HLA loci as HLA loci possibly associated with other autoimmune diseases. We started our further investigation from FBW7 located on chromosome 4, encoding an E3 ubiquitin ligase that targets key regulators of cell division and growth for ubiquitylation and subsequent degradation by the proteasome [24]. FBW7 suppresses colorectal cancer [25, 26], gastric cancer [27], and pancreatic cancer [28] through ubiquitin degradation of varied components in the pathway. The experimental data obtained from our study validated that FBW7 was downregulated in T1D mouse model. Growing evidence has implied that F-box protein protects pancreatic beta cells and negatively regulates the cytokines in renal mesangial cells [16, 29]. Results in the present study confirmed that FBW7 impeded the T1D incurrence, corresponding to downregulated cytokines expression and inhibited pancreatic beta cell apoptosis, indicating its significance in autoimmune diseases.

A wide variety of oncogenic proteins are found to be the substrates of FBW7 for ubiquitination including c-Myc [30], SREBP [31], and hypoxia inducible factor-1α [26], many of which play key roles in metabolic pathways [32]. We found EZH2 was the FBW7 substrate as we investigated the downstream pathway. Similarly, FBW7-dependent ubiquitination and degradation of EZH2 is found in the pancreatic cancer cells and prevents cancer migration and invasion [11]. EZH2 degradation by FBW7 is meanwhile reported in macrophages [33]. These results strongly suggest the important function of FBW7 in the regulation of the EZH2 protein level.

As the enzymatic subunit of polycomb repressive complex 2, EZH2 epigenetically silences the genes in target promoters by H3K27me3 [34]. In addition to H3K27me3, EZH2 can also bind DNA methyltransferase 1 (DNMT1) thus methylating DNA [35]. Kowluru et al. reported that DNMT1 alters the methylation status of matrix metalloprotein-9 promoter in diabetes [36, 37]. Studies on the parallel possible methylation pathway, as well as determination of 5mC and 5hmC levels at the ZBTB16 promoter, are currently ongoing in our lab.

ZBTB16 has been proved to be beneficial for tumor suppression as it represses c-myc oncogene [38]. ZBTB16 is critical in diabetes progression. Activation of ZBTB16 reduces serum advanced glycation end (AGE) products in STZ-induced diabetic mice and further inhibits AGE-induced apoptosis [39]. Furthermore, ZBTB16 involves in insulin signaling pathway regulation by reduction of phosphorylation levels of IRS1, Ake, and FoxO1 in the mouse model [40]. In our study, we described ZBTB16 negatively correlated to pancreatic beta cell apoptosis and contributed to beta cell viability. Therefore, the downstream pathway of T1D inhibition by FBW7 has been established.

**Fig. 6:**
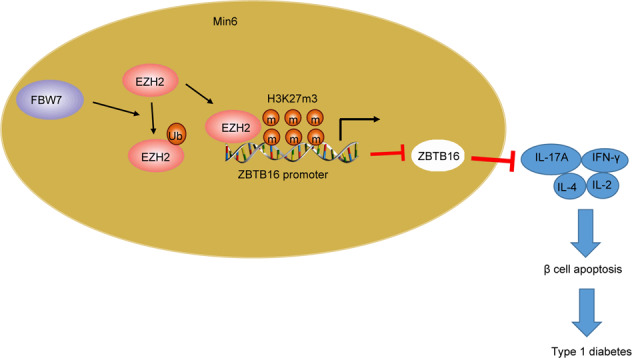
The mechanism graph of the regulatory network and function of FBW7. E3 ubiquitin ligase FBW7 prevents T1D by upregulating ZBTB16 expression via histone methyltransferase EZH2.

Taken together, this study suggests the role of FBW7 (Fig. 6), which sits outside the HLA region, in the prevention of T1D occurrence. We established the FWB7/EZH2/ZBTB16 axis in the T1D pathogenesis and provided a new therapeutic target for T1D treatment. In addition, cell lines with low FBW7 and EZH2 endogenous levels will be included in our further research to study the effect of overexpression without the interference of endogenous proteins. Also, silencing experiments are required to associate with overexpression experiments to consolidate our thesis in the future.

## Materials and methods

### Ethics statement

Animal experiments were approved by the Ethics Committee of the Second Hospital of Jilin University and conducted in strict accordance to *the Guide for the Care and Use of Laboratory Animals* published by the US National Institutes of Health.

### Bioinformatics analysis

T1D-related gene expression microarray data GSE29142 was obtained through the GEO database (https://www.ncbi.nlm.nih.gov/gds), and differential analysis was performed by using the “limma” software package in the R language. The microarray data contains 19 samples, including 10 normal samples and 9 T1D samples. Using ∣logFC∣ > 0.5, *p* < 0.05 as the threshold, the significantly differentially expressed genes were screened.

### Establishment of mouse model

A total of 70 male non-obese diabetic mice aged 4 weeks were obtained from Shanghai Eason Biotech Co., Ltd. (Shanghai, China). The mice were randomized into the normal (*n* = 10) and T1D model groups (*n* = 60). For the STZ-induced T1D group, STZ (Sigma-Aldrich, Shanghai, China) at a dose of 40 mg/kg was administered intraperitoneally for 6 successive days. Blood glucose concentrations were measured using an OneTouch Ultra 2 blood glucose meter (LifeScan Inc., Milpitas, CA, USA) every other day. When two successive blood glucose readings were more than 250 mg/dL, the mouse was considered as diabetic [41]. Afterwards, the occurrence was recorded every 6 days. The presented data were cumulative incidence of T1D collected from 3 independent experiments.

### Vector construction and cell transfection

FBW7 and the EZH2 coding region of the gene were ligated into the pLVX-DsRed2-3FLAG vector. GFP Lentivirus control plasmid (Heyuan, Shanghai, China) was applied in this study. FBW7 shRNA, ZBTB16 shRNA, and NC shRNA were constructed by Shanghai Gene Pharma Co., Ltd. (Shanghai, China). The 293T cells were cultured and inoculated into a Petri dish 1 h prior to transfection. Lentiviral vectors containing pLVX-FBW7 and pLVX-EZH2 were transfected to 293T cells through lipofectamine2000.

After 24 h, the cell medium was changed to Dulbecco’s modified Eagle medium (DMEM) supplemented with 10% fetal bovine serum (FBS). After 48 h, the cells were centrifuged and the supernatant was collected, filtered using a 0.45 μm polyvinylidene fluoride (PVDF) filter, and kept at −80 °C. Cells were transfected with the following plasmids: shFBW7-1, OE-FBW7, OE-EZH2, sh-ZBTB16 as well as the corresponding controls.

Lentiviral vectors with FBW7 overexpression, ZBTB16 interference, and control vectors were transduced into T1D mice via tail vein injection. The occurrence of T1D was determined after 24 h. The mice were sacrificed by cervical dislocation after 30 days. The pancreatic tissues, splenocytes, and pancreatic islet beta cells of mice were isolated [42]. Islet autoantigen glutamic acid decarboxylase (GAD65, 20 μg/mL) was added to the splenocytes in vitro in order to induce a T1D-specific T-cell response.

### Isolation of mouse islets

Islets were isolated from the pancreas as follows. Briefly, the distal end proximal to the duodenum was occluded, and 4 mL collagenase P solution (1 mg/mL) was slowly injected into the common bile duct. The distended pancreas was excised and digested in a water bath at 37 °C for 30 min. Then, collagenase digest was subjected to a Ficoll gradient separation to promote the harvest of islets. Subsequently, the islets were counted under an inverted microscope.

### ELISA

To determine the cytokine levels, splenocytes were cultured in DMEM supplemented with 10% FBS (0.2 mL) at a final concentration of 1.5 × 10^6^ cells/well. The supernatant was collected after 48 h and cytokines, IL-17A (M1700), IL-2 (M2000), IL-4 (M4000B), and IFN-γ (MIF00), were assessed using a quantitative ELISA kit (R&D Systems, Inc., Minneapolis, MN, USA) [43].

### RT-qPCR

Total RNA was isolated using TRIzol (Invitrogen, Carlsbad, CA, USA) and reversely transcribed to cDNA using PrimeScript™ RT Reagent Kit with gDNA Eraser (RR047A, Takara, Kusatsu, Japan). After an RT step at 37 °C for 15 min followed by denaturation at 85 °C for 5 s, cDNA was mixed with TB Green Premix Ex Taq kit (RR420A, Takara). Real-time PCR was conducted in the ABI7500 system (Applied Biosystems, Foster City, CA, USA). Each sample was analyzed in triplicate. FBW7, EZH2, and ZBTB16 primer sequences were synthesized by Shanghai Sangon Biotech Co., Ltd (Supplementary Table 1). CT values were recorded and glyceraldehyde-3-phosphate dehydrogenase (GAPDH) was served as the internal reference for FBW7, EZH2, and ZBTB16. The 2^-ΔΔCT^ method was applied to calculate the relative expression of genes.

### Western blot assay

Tissue or cell total protein was extracted using Radio Immunoprecipitation Assay lysis buffer (R0010, Solarbio, Beijing, China) at 4 °C for 15 min followed by centrifugation at 15,000 r/min for 15 min. The protein concentration was measured using a bicinchoninic acid kit (20201ES76, Yeasen, Shanghai, China). Then, the proteins were separated by sodium dodecyl sulfate–polyacrylamide gel electrophoresis, transferred onto the PVDF membrane, and blocked in 5% bovine serum albumin for 1 h. Next, the membrane was incubated at 4 °C overnight with diluted primary antibodies: Fbxw7 (ab109617, 1: 200), EZH2 (ab186006, 1: 500), ZBTB16 (ab39354, 1: 1000), FLAG (ab1162, 1: 200), HA (ab9110, 1:2000), Myc (ab9106, 1:500), GAPDH (ab8245, 1:1000), and Ub (ab7780, 1:500). All the rabbit polyclonal antibodies were purchased from Abcam (Cambridge, UK) unless otherwise noted. After incubation, the membrane was re-probed with horseradish peroxidase-labeled anti-Rabbit immunoglobulin G (IgG) H&L (ab205718, 1:10,000) for 1 h at room temperature. The blots were visualized with enhanced chemiluminescence regents. The images were captured using FUSION FX5 (Vilber Lourmar, France), and analyzed by ImageJ 1.48u (National Institutes of Health, USA). GAPDH (ab9485, 1:500) was served as the internal reference.

### ChIP assay

ChIP assay was performed to determine the accumulation of EZH2 and H3K27me3 in the ZBTB16 promoter region using the EZ-Magna ChIP kit (17-10086, Millipore, Billerica, MA, USA). Airway smooth muscle cells in the logarithmic growth phase were crosslinked with 1% formaldehyde for 10 min. Crosslinking was quenched by the addition of 125 mM glycine at room temperature for 5 min. Cells were sheared to 200–1000 bp by sonication and centrifuged at 14,000 × *g* at 4°C for 10 min. The supernatant (100 μL), ChIP dilution buffer (900 μL), protease inhibitor cocktail (50×, 20 μL), and Protein A-Agarose/Salmon Sperm DNA (60 μL, Sigma-Aldrich) were mixed at 4 °C for 1 h and allowed to stand still at same temperature for 10 min. The supernatant (20 μL) from the experimental group was mixed with EZH2 Ab (1 μL, ab186006, 1:200), H3K27me3 Ab (1 μL, ab192985, 1:200, Abcam), and Protein A-Agarose/Salmon Sperm DNA (60 μL). For the control experiments, rabbit IgG (1 μL, ab172730, 1:200, Abcam) was used. Then, the precipitate was washed with low salt buffer (1 mL), high salt buffer (1 mL), LiCl solution (1 mL), and TE buffer (1 mL, 2×). ChIP wash buffer (250 μL, 2×) was added to elute the complex. DNA was de-crosslinked and recycled by adding NaCl (5 M, 20 μL) in which RT-qPCR was performed for further determination [18].

### Co-IP

IP was carried out with Pierce™ Co-Immunoprecipitation Kit (Thermo Fisher Scientific, Waltham, MA, USA) with 5 μg of anti-EZH2 or anti-IgG antibody as previously published method [44].

### Protein stability assay

Vector shControl and shFBW7 were transfected into Min6 cells per calcium phosphate method. After 24 h, the cells were treated with CHX (CalBiochem, Gibbstown, NJ, USA), an inhibitor of eukaryotic translation, at a dose of 40 μg/mL. The cells were harvested and lyzed at designed time points. The lysate was analyzed by Western blot, imaged, and quantified by Licor Odyssey and Image Quant software [45, 46].

### Flow cytometry

Pancreatic beta cells were isolated from T1D mice and cultured in the medium supplemented with TNF-α (10 ng/mL) for 3 days to simulate the insulin-dependent T1D [42]. After removal of medium, the cells were washed twice with PBS and centrifuged. The cell pellet was resuspended in the binding buffer to prepare a single cell suspension with a concentration of 5 × 10^5^ cell/mL. Annexin V (5 μL) and 7-aminoactinomycin D (5 μL) were added to the suspension at room temperature protected from light. The mixture was incubated for 30 min followed by determination using flow cytometry (BD Bioscience, San Jose, CA, USA).

### Assessment of cytotoxicity by cell counting kit-8 (CCK-8) assays

Cells were treated with the same protocol as stated in the flow cytometry. Cytotoxicity was checked using the CCK-8 kit (Yisen, Shanghai, China). First, the cell suspension was placed in a 96-well plate with a concentration of 5 × 10^4^ cells/well, and pre-warmed in the incubator (37°C, 5% CO_2_). Next, CCK-8 solution (10 μL) was added to each well and the plate was incubated for 1–4 h. Finally, the absorbance at 450 nm was recorded on a microplate reader (Bio-Rad, Hercules, CA, USA) [43].

### Assessment of insulin secretion

Min6 and pancreatic beta cells were inoculated on a 24-well plate at the concentration of 5 × 10^4^ cell/well pretreated with or without Notoginsenoside R1. Next, the pancreatic beta cells were placed in the medium supplemented with TNF-α (20 ng/mL) for 3 days to simulate the islet inflammation in T1D. The supernatant was collected and the insulin concentration was determined by radioimmunoassay [42, 47].

### IHC

The paraffin-embedded tissue sections were deparaffinized and hydrated, followed by suppression of endogenous peroxidase activity by incubating in 0.3% H_2_O_2_ for 30 min at 37 °C. After PBS washing, the tissue sections were boiled in 10 mmol/L citrate buffer (pH 6.0) at 100 °C for 30 min, blocked with 5% normal goat serum at 37 °C for 1 h, and then incubated with FBW7 antibody (ab109617, 1:200, Abcam, Cambridge, UK) at 4 °C overnight. After that, the tissue sections were incubated with secondary antibody IgG (ab205718; 1:2000) at 37 °C for 1 h and then with horseradish peroxidase-conjugated streptavidin (1:1000 dilution) at 37 °C for 45 min, followed by treatment of newly prepared DAB for color development. All tissue sections were counterstained with hematoxylin. Finally, the stained tissue sections were analyzed under the OLYMPUS BX51 microscope.

### Statistical analysis

Statistical and mathematical data were processed by SPSS 21.0 (IBM Corp., Armonk, NY, USA). Values were summarized as mean ± standard deviation. Data comparisons between two groups were analyzed by unpaired *t*-test while that among multiple groups were performed by one-way analysis of variance (ANOVA). Statistical analysis with time-based measurements within each group was performed using repeated measures ANOVA. A value of *p* < 0.05 indicated a significant difference.

## Supplementary information

Supplementary Table 1

## Data Availability

The datasets used and/or analysed during the current study are available from the corresponding author on reasonable request.
