# Supplementary material for: Suppressive role of E3 ubiquitin ligase FBW7 in type I diabetes in non-obese diabetic mice through mediation of ubiquitination of EZH2
Source: Cell Death Discov. 2021 Nov 20;7:361. doi: 10.1038/s41420-021-00605-x (PMC8606006; doi:10.1038/s41420-021-00605-x)
Supplement: Supplementary file 1 — Supplementary Table 1 [file 41420_2021_605_MOESM1_ESM.doc]

**Supplementary** Table 1 Primer sequences for RT-qPCR

| Gene of interest | Sequence |
| --- | --- |
| FBW7 (human) | Forward: 5'-CCACTGGGCTTGTACCATGTT-3' |
|  | Reverse: 5'-CAGATGTAATTCGGCGTCGTT-3' |
| FBW7 (mice) | Forward: 5'-CGAGACTTCATCTCCTTGCTTCC-3' |
|  | Reverse: 5'-CCAGAGAAGGTTATCCTCAGCC-3' |
| EZH2 (human) | Forward: 5'-GCCAGACTGGGAAGAAATCTG-3' |
|  | Reverse: 5'-TGTGCTGGAAAATCCAAGTCA-3' |
| EZH2 (mice) | Forward: 5'-CAACCCGAAAGGGCAACAAA-3' |
|  | Reverse: 5'-TCACCAGTCTGGATAGCCCT-3' |
| ZBTB16 (human) | Forward: 5'-GAGCTTCCTGATAACGAGGCTG-3′ |
|  | Reverse: 5'-AGCCGCAAACTATCCAGGAACC-3′ |
| ZBTB16 (mice) | Forward: 5'-ACATACGGGTGTGAACTCTGCG-3′ |
|  | Reverse: 5'-TGAGAACTGGGCACCGCATTGA-3′ |
| GAPDH (human) | Forward: 5'-CACCCACTCCTCCACCTTTG-3′ |
|  | Reverse: 5'-CCACCACCCTGTTGCTGTAG-3′ |
| GAPDH (mice) | Forward: 5'-CATCACTGCCACCCAGAAGACTG-3′ |
|  | Reverse: 5'-ATGCCAGTGAGCTTCCCGTTCAG-3′ |

Note: RT-qPCR, reverse transcription quantitative polymerase chain reaction; FBW7, F-box and WD repeat domain-containing 7; EZH2, enhancer of zeste homolog 2; ZBTB16, Zinc finger and BTB domain containing 16; GAPDH, glyceraldehyde-3-phosphate dehydrogenase.
